# Characteristics of 29 novel atypical solute carriers of major facilitator superfamily type: evolutionary conservation, predicted structure and neuronal co-expression

**DOI:** 10.1098/rsob.170142

**Published:** 2017-09-06

**Authors:** Emelie Perland, Sonchita Bagchi, Axel Klaesson, Robert Fredriksson

**Affiliations:** 1 Molecular Neuropharmacology, Department of Pharmaceutical Biosciences, Uppsala University, Uppsala, Sweden; 2 Pharmaceutical Cell Biology, Department of Pharmaceutical Biosciences, Uppsala University, Uppsala, Sweden

**Keywords:** major facilitator superfamily, solute carrier, atypical SLC, family clustering, topology, nutrition

## Abstract

Solute carriers (SLCs) are vital as they are responsible for a major part of the molecular transport over lipid bilayers. At present, there are 430 identified SLCs, of which 28 are called atypical SLCs of major facilitator superfamily (MFS) type. These are MFSD1, 2A, 2B, 3, 4A, 4B, 5, 6, 6 L, 7, 8, 9, 10, 11, 12, 13A, 14A and 14B; SV2A, SV2B and SV2C; SVOP and SVOPL; SPNS1, SPNS2 and SPNS3; and UNC93A and UNC93B1. We studied their fundamental properties, and we also included CLN3, an atypical SLC not yet belonging to any protein family (Pfam) clan, because its involvement in the same neuronal degenerative disorders as MFSD8. With phylogenetic analyses and bioinformatic sequence comparisons, the proteins were divided into 15 families, denoted atypical MFS transporter families (AMTF1-15). Hidden Markov models were used to identify orthologues from human to *Drosophila melanogaster* and *Caenorhabditis elegans*. Topology predictions revealed 12 transmembrane segments (for all except CLN3), corresponding to the common MFS structure. With single-cell RNA sequencing and *in situ* proximity ligation assay on brain cells, co-expressions of several atypical SLCs were identified. Finally, the transcription levels of all genes were analysed in the hypothalamic N25/2 cell line after complete amino acid starvation, showing altered expression levels for several atypical SLCs.

## 1. Introduction

It is essential that transport of nutrients, waste and drugs over lipid bilayers is executed accurately to keep the homeostasis within the body, and disturbances in the transport systems are associated with Mendelian diseases [1,2]. Most transport is carried out by three major types of transporters [3]: channels, primary active transporters and secondary active transporters. With its 430 members [4], the secondary active transporters, commonly called the solute carriers (SLCs), constitute the largest group of membrane-bound transporters in humans [5]. The SLCs are currently divided into 52 families [6]. SLCs use energy from coupled ions or facilitative diffusion to move substrates via coupled transport, exchange or uniport [7]. SLC transporters are crucial throughout the body, and their importance is particularly prominent in the brain, where they, for example, gate nutrients over the blood–brain barrier [8], terminate neuronal transmission by clearing neurotransmitters from the synaptic cleft [9,10], refill vesicles [11] and maintain the glutamine–glutamate cycle [12]. These mechanisms are used in pharmacology, where transporters are used either as direct drug targets [2,10] or indirectly as facilitators of drug distribution to specific tissues [13].

Most SLC proteins can be divided into Pfam clans based on sequence similarity [4,14], where the major facilitator superfamily (MFS; Pfam clan id: CL0015), amino acid/polyamine/organocation (APC; CL0062), cation : proton antiporter/anion transporter (CPA/AT; CL0064) and drug/metabolite transporter superfamily (DMT; CL0184) clans include more than one SLC family [4,14,15]. Approximately one-third of all SLCs belong to the MFS clan [4], making it the largest group of phylogenetically related SLCs. MFS is a large and diverse family of proteins [16], which evolved from a common ancestor [17]. This ancient family has members in several organisms, including bacteria, yeast, insects and mammals [16–20]. As MFS proteins are closely related, they usually share protein topology. MFS proteins are single polypeptides [16], usually composed of 400–600 amino acids [21]. They probably arose by duplication of a six transmembrane segment (TMS), providing the N and C domains, which are connected by a long cytoplasmic loop between TMS 6 and 7 [21], resulting in a 12 TMS protein [17]. It is suggested that transporters containing the MFS fold move substrates via the rocker-switch mechanism [22] or through the updated clamp-and-switch model [23].

Among the 430 human SLCs, 30 proteins are called atypical SLCs as they are evolutionarily connected to SLCs [4], but are yet to be classified into any existing SLC family. Twenty-eight of the atypical SLCs belong to the MFS Pfam clan [4] and are discussed in this article, together with the non-MFS Pfam clan protein ceroid lipofuscinosis, neuronal 3 (CLN3). According to the transporter classification database [24], CLN3 belongs to the equilibrative nucleoside transporter, which is a subfamily of the larger MFS superfamily. Additional atypical SLCs are TMEM104 that belong to the APC clan and OCA2 which cluster with the IT clan [4]. The atypical SLCs of MFS type are the major facilitator superfamily domain containing (MFSD) proteins, MFSD1, 2A, 2B, 3, 4A, 4B, 5, 6, 6 L, 8, 9, 10, 11, 12, 13A, 14A and 14B; the synaptic vesicles glycoprotein 2 (SV2) proteins, SV2A, SV2B and SV2C; the SV2-related proteins SVOP and SVOPL; three sphingolipid transporters, SPNS1, SPNS2 and SPNS3; and two unc-93 proteins, UNC93A and UNC93B1 [4]. These proteins were identified as possible SLCs by searching the human proteome using hidden Markov models (HMM) composed of known SLC sequences originating from the MFS Pfam clan [4]. MFSD7 was also included in the analysis and considered as an atypical SLC, because of its status as an orphan protein. However, MFSD7 is already classified into the SLC49 family [25]. Knowledge about atypical SLCs is limited, which is why we aim to present a cohesive study of the basic characteristics of 29 atypical SLCs belonging to the MFS clan. They cluster phylogenetically with SLC families from the MFS Pfam clan, SLC2, 15 16, 17, 18, 19, SLCO (SLC21), 22, 29, 33, 37, 40, 43, 45, 46 and 49 [4], suggesting that they have transporter properties, and are involved in homeostatic maintenance. Since the atypical SLCs are MFS proteins, it is likely that they all are constituted of the common 12 TMS polypeptides [17], which has been predicted for some (e.g. MFSD1 [19], MFSD2A [26], MFSD8 [27,28], SVOP [29] and UNC93B1 [30]), while CLN3 only has six predicted TMSs [31,32].

Several atypical SLCs are expressed in the brain, where they are found in neurons [19,20,33,34] and the CNS vasculature system [35]. Concerning their subcellular expression, atypical SLCs are expressed both in the plasma membrane [19,36] and intracellular membranes [27,33,37–40] (localizations summarized in table 1). There are also contradictory reports, suggesting that the same protein is located in several subcellular locations; MFSD1 is found in embryonic mouse neuronal plasma membranes [19] and lysosomal membranes in HeLa and rat liver cells [39,41], which could be explained by translocation of the transporters in the cell, serving multiple functions under different conditions or states of the cell. SV2 proteins are identified both at synaptic vesicles [49] and the plasma membrane, possibly because the synaptic vesicles fuse with the plasmalemma during neurotransmitter release. CLN3 is expressed at the plasma membrane as well as on endosome/lysosome membranes [34], where it is involved in neuronal ceroid lipofuscinosis, which leads to neurodegenerative disorders resulting from the accumulation of lipofuscin [57]. This is of interest because MFSD8 (known as CLN7) is also involved in this pathology [58].

**Table 1.** Basic facts about atypical SLCs.

atypical SLC	aliases	Human Genome Nomenclature Committee (HGNC) ID TCDB ID	protein size	subcellular expression	substrate
MFSD1	SMAP4	HGNC:25874	514aa	plasma membrane [19] and lysosomes [39,41]	
MFSD2A	NLS1	HGNC:25897 TC: 2.A.2.3.8	543aa	plasma membrane [42] and ER [37]	sodium-dependent phospholipid transport [43]
MFSD2B		HGNC:37207	504aa	ER [37]	
MFSD3		HGNC:25157 TC: 2.A.1.25.4	412aa	plasma membrane [19]	
MFSD4A	MFSD4	HGNC:25433	514aa		
MFSD4B	KIAA1919, NAGLT1	HGNC:21053	518aa	intracellular [44,45]	sodium-dependent glucose transport [38]
MFSD5	hsMOT2	HGNC:28156	557aa		molybdate-anions transport [46]
MFSD6	MMR2	HGNC:24711 TC: 2.A.1.65.6	791aa		
MFSD6 L		HGNC:26656 TC: 2.A.1.65.10	586aa		
MFSD7	MYL5, SLC49A3	HGNC:26177 TC: 2.A.1.28.2	559aa		
MFSD8	CLN7	HGNC:28486 TC: 2.A.1.2.56	518aa	lysosomal [27]	
MFSD9		HGNC:28158 TC: 2.A.1.2.72	474aa		
MFSD10	TETRAN	HGNC:16894 TC: 2.A.1.2.73	455aa	plasma membrane [47] and intracellular [36]	organic anions [36]
MFSD11		HGNC:25458 TC: 2.A.1.58.3	449aa		
MFSD12		HGNC:28299	480aa	mitochondria [44,45]	
MFSD13A	TMEM180	HGNC:26196	517aa		
MFSD14A	HIAT1, MF14A	HGNC:23363	490aa	intracellular [33]	presumed sugar transport [48]
MFSD14B	HIATL1, MF14B	HGNC:23376 TC: 2.A.1.2.30	506aa	intracellular [33]	
SV2A	KIAA0736	HGNC:20566	742aa	vesicular [49–51]	sugar [52]
SV2B	KIAA0735	HGNC:16874	683aa	vesicular [51]	
SV2C	KIAA1054	HGNC:30670	727aa	vesicular [51,53]	
SVOP		HGNC:25417 TC: 2.A.1.82.3	548aa	vesicular [54,55]	
SVOPL		HGNC:27034	492aa		
SPNS1	SPIN1, HSpin1	HGNC:30621 TC: 2.A.1.49.2	528aa	mitochondria [56]	
SPNS2		HGNC:26992 TC: 2.A.1.49.6	549aa		
SPNS3		HGNC:28433	512aa		
UNC93A		HGNC:12570 TC: 2.A.1.58.2	457aa		
UNC93B1	UNC93, UNC93B	HGNC:13481 TC: 2.A.1.58.7	597aa	ER [40]	
CLN3	BTS, Battein	HGNC:2074 TC: 2.A.57.5.1	438aa	plasma membrane [34] and lysosomal [32,34]	

Several atypical SLCs are affected by food intake and nutritional status, where both high-fat diet and food deprivation alter their expression levels in rodents [19,20,33,37,59]. Furthermore, the expression of *Mfsd11* is altered in immortalized mouse hypothalamic N25/2 cells exposed to complete amino acid starvation [60]. This suggests that the atypical SLCs are involved in maintaining the nutritional status both *in vivo* and *in vitro*, which reinforces the importance of understanding their fundamental properties.

Here, we phylogenetically studied interrelations between the atypical SLCs of MFS type and similarities between the protein sequences. Furthermore, we investigated if the atypical SLCs met the requirements to belong in any of the existing 52 SLC families. SLC families are divided on the basis of homology or phenotype [61], and a protein must share at least 20% sequence identity to another family member [62] to be placed in that family. HMMs were built to search proteomes from several organisms to identify related proteins, showing their evolutionary development. Furthermore, topology predictions were made for the human protein sequences, suggesting 12 TMS for all investigated atypical SLCs, except for CLN3 with its 11 predicted TMS. With single-cell RNA sequencing data retrieved from 10X genomics (www.10xgenomics.com/), we examined which atypical SLCs were expressed in the same cell from an 18 days mouse embryo brain. We supplemented these results at protein level using *in situ* proximity ligation assay [63,64], where interaction between proteins were quantified in mouse brain sections. Finally, using microarray data [60], we analysed if and how the atypical SLCs were affected by complete amino acid deprivation in N25/2 cells.

## 2. Material and methods

### 2.1. Clustering of human atypical SLCs of MFS type

To study the interrelations between atypical SLCs of MFS type, the longest amino acid sequences for the human MFSD1, 2A, 2B, 3, 4A, 4B, 5, 6, 6 L, 7, 8, 9, 10, 11, 12, 13A, 14A, 14B, SV2A, SV2B, SV2C, SVOP, SVOPL, SPNS1, SPNS2, SPNS3, UNC93A, UNC93B1 and CLN3 proteins (for sequences, see electronic supplementary material, table S1) were combined in a multiple PSI/TM tcoffee sequences alignment [65] before inferring their relationship according to the Bayesian approach, as implemented in MrBayes 3.2.2 [66,67]. The analysis was run via the Beagle library [68] on six chains (five heated and one cold), with two runs in parallel (*n* runs = 2) for a maximum of 2 000 000 generations.

An additional tree was built, including all known SLC and atypical SLC sequences originating from the MFS Pfam clan. After a multiple PSI/TM tcoffee sequence alignment [65], a phylogenetic tree was built using RAxML [69] on a 14 Core Intel CPU workstation. The tree was calculated on protein sequences using the GAMMAJTT amino acid model with 500 bootstrap replicas, and a consensus tree was calculated from these using the built in consensus tree calculation in RAxML.

SLC families are built on homology, function, phenotype [61] and sequence identities [62]. As the atypical SLCs group among SLC families [4], it is possible that they belong to already annotated SLC or new families. To study this further, sequence identities were analysed using global pairwise sequence alignment based on the Needleman–Wunsch algorithm [70]. The similarities between human atypical SLCs were analysed, followed by comparison with all SLC members of MFS type (SLC family 2, 15 16, 17, 18, 19, SLCO, 22, 29, 33, 37, 40, 43, 45, 46 and 49) (matrixes in electronic supplementary material, table S1). To group the atypical proteins into families, the following parameters were considered: (i) 20% identity to other atypical SLCs, (ii) phylogenetic clustering among the atypical SLCs, (iii) phylogenetic clustering among SLCs and (iv) 20% identity to at least one other SLC family member. Families including atypical SLCs were called atypical MFS transporter families (AMTF).

### 2.2. Hidden Markov models to identify related proteins

Hidden Markov models (HMM) were built for all 29 atypical SLCs by running mammalian sequences through HMMbuild from the HMMER package [71]. The models were used to search the protein datasets (obtained from Ensembl version 86 [72]) listed in table 2, to identify related proteins in yeast, roundworm, fruit fly, zebrafish, chicken, mouse and human. Sequences were manually curated, and proteins originating from the same locus and pseudogenes were removed. Genes not in closest phylogenetic proximity with the human version were also removed, as they were either without specific orthologues in mammals or that they phylogenetically clustered to other proteins. Predicted full-length proteins were kept as related reliable hits. As the atypical SLCs are relatively similar in amino acid sequence, proteins were identified in several HMM. Phylogenetic analyses were therefore performed, using RAxML, as described above, to determine which were orthologues and other related proteins. All identified proteins were annotated and listed with accession number in electronic supplementary material, table S2. Note that some proteins were given names with Like (L) as a suffix, and these were related proteins identified by the HMM, without belonging to the human protein cluster. It is possible that these are orthologues to proteins not studied here, or that they lack equivalents in humans.

**Table 2.** Datasets searched for related proteins.

species	common name	dataset version
*S. cerevisiae*	yeast	R64-1-1.pep.all
*C. elegans*	roundworm	WBcel235.pep.all
*D. rerio*	zebrafish	GRCz10.pep.all
*D. melanogaster*	fruit fly	BDGP6.pep.all
*G. gallus*	chicken	Galgal4.pep.all
*H. sapiens*	human	GRCh38.pep.all
*M. musculus*	mouse	GRCm38.pep.all

### 2.3. Structural predictions to study possible transporter properties

For a MFS protein to have optimal transporter properties, 12 transmembrane segments (TMS) are required [17]. To investigate if the proteins of interest possessed the common MFS structures, topology predictions were done using the constrained consensus TOPology prediction server (CCtop) [73,74]. CCtop combine the results from 10 known online topology tools to incorporate parameters like hydrophobicity, charge bias, helix lengths and signal peptides in the predictions [75,76], and further combine the result with structural information from existing experimental and computational sources [73]. Three of the proteins were not predicted to contain 12 TMS, MFSD13A, SPNS3 and CLN3, and homology models were built to verity these three predictions. The tertiary structures were built using Swiss Model, a fully automated homology program [77], where structurally known MFS transporters were used as templates. MFSD13A was aligned against the bacterial sodium symporter, MelB [78], providing global model quality estimation (GMQE) of 0.47. GMQE indicates the reliability of models on a scale range from 0 to 1, where 1 represents total reliability. For the SPNS3 model, the proton-driven YajR transporter from *E. coli* was used as template [79], with a GMQE of 0.45. For CLN3, a peptide MFS transporter from bacteria [80] was used as template, providing a score of 0.44. Homology models were adjusted in the open-source Java viewer Jmol [81] (http://www.jmol.org/). Finally, the amino acids in each TMS from the homology models were manually identified and compared with the ones predicted by CCtop.

### 2.4. RNA analysis from single brain cells, to identify co-expression between atypical SLCs

The complete dataset (9 k brain cells from an E18 Mouse) for single-cell RNA sequencing from E18 mouse brain was downloaded from 10X Genomics (www.10xgenomics.com) under a Creative Commons license. The data was analysed to investigate co-expression of atypical SLCs of MFS type in single brain cells. Of note, 10 289 cells were collected from cortex, hippocampus and subventricular zone of an E18 mouse, and sequenced on Illumina Hiseq4000 with approximately 42 000 reads per cell (10X Genomics). A digital expression matrix was constructed based on that data to extract information from the atypical SLCs, and removing cells with fewer than three identified transcripts. Then, cells expressing fewer than two different atypical SLC transcripts were removed. This resulted in 9693 cells co-expressing 21 atypical SLCs. To assess the significance of these observations, we used a bootstrapping approach, implemented in a custom written Java program. Briefly, in the implementation, as our null hypothesis, we assumed that there was no co-expression observed in the data over what is expected by chance. We created a dataset with the same frequency of each of the transcripts as observed in our actual data and randomly assigned these transcripts to 9693 cells. This process was repeated 1000 times and the mean number of transcripts and the population standard deviation of the number of transcripts for each cell were calculated. We considered any values one standard deviation above and below the mean of the bootstrapped data as significantly different from true chance.

### 2.5. *In situ* proximity ligation assay, sample preparation, execution and analysis

To complement the co-expression, *in situ* proximity ligation assay (PLA) was performed. Intra-peritoneal injections of sodium Pentobarbital (Apoteket Farmaci, Sweden) (10 mg kg^−1^) were used to anesthetize adult C57BL6/J mice, followed by trans-cardiac perfusion using 4% formaldehyde (Histolab) and then paraffin embedding, as described in [20]. The brains were cut in 7 µm sections using a Microm 355S STS cool cut microtome and attached on Superfrost Plus slides (Menzel-Gläser). Each slide was dried overnight at 37°C before stored at 4°C.

Sections were deparaffinized by 10 min washes in X-TRA solve (Medite, Dalab), followed by an ethanol (Solveco) rehydration series ranging from 100% to water. Antigen retrieval was performed in boiling 0.01 M citric acid (Sigma-Aldrich) at pH 6.0, for 10 min, after which the slides were cooled, washed in PBS, and placed in a humidity chamber throughout the experiment to avoid drying out during incubations at 37°C. Brain sections were blocked for 1 h at 37°C in blocking solution, provided by Duolink II fluorescence kit (orange detection reagents; Olink Biosciences), followed by primary antibody incubation at 4°C overnight (table 3 for antibody information). The antibodies were diluted in specific antibody diluent provided by Duolink II fluorescence kit (orange detection reagents; Olink Biosciences). The slides were then washed 2 × 5 min in wash buffer A, while kept on orbital shaking. Two PLA probes, PLUS and MINUS, were added to each selected primary antibody combinations (summarized in table 3). The probes were diluted in antibody diluent, and added to the slides followed by incubation for 1 h at 37°C. Slides were washed for 2 × 5 min in Wash buffer A, before adding the Ligation-Ligase solution (Duolink II fluorescence kit; Olink Biosciences), followed by 30 min incubation at 37°C. Slides were washed 2 × 5 min in Wash Buffer A, before adding the Amplification-Polymerase solution (Duolink II fluorescence kit; Olink Biosciences), followed by incubation for 100 min, at 37°C. After tapping the Amplification-Polymerase off, the slides were washed 2 × 10 min in Wash Buffer B, followed by a 1 min washing step using 0.01× Wash Buffer B. Slides were dried under dark conditions, and mounted in Duolink *in situ* Mounting Medium, including DAPI (Olink Biosciences).

**Table 3.** Antibody combinations and concentrations used for the *in situ* proximity ligation assay.

protein	origin	concentration	supplier	catalogue number	combined with	PLA probes
MFSD3	rabbit	1 : 50	Sigma-Aldrich	AV51707	MFSD11	+
MFSD4A	rabbit	1 : 50	Sigma-Aldrich	SAB1305276/AV53395	MFSD11	+
MFSD6	goat	1 : 20	Sigma-Aldrich	SAB2502050	MFSD11	−
MFSD7	rabbit	1 : 100	Abcam	ab180496	MFSD11	+
MFSD8	rabbit	1 : 50	Sigma-Aldrich	HPA044802	MFSD9 MFSD11	+
MFSD9	goat	1 : 50	Santa Cruz	sc-247973	MFSD8 MFSD10 MFSD14A MFSD14B	−
MFSD10	rabbit	1 : 20	Sigma-Aldrich	HPA037398	MFSD9 MFSD11	+
MFSD11	goat/rabbit	1 : 80	Santa Cruz/Sigma-Aldrich	sc-243472/HPA022001	MFSD3 MFSD4A MFSD6 MFSD7 MFSD8 MFSD10 MFSD14a	−/+
MFSD14A	rabbit	1 : 100	Sigma-Aldrich	SAB1306449	MFSD9 MFSD11	+
MFSD14B	rabbit	1 : 100	Sigma-Aldrich	SAB2107506	MFSD9 MFSD11	+

Micrographs were taken using a Zeiss Axioplan 2 epifluorescent microscope, and 11 Z-stacks from various brain areas, like cortex and striatum, were acquired for each antibody-pair combination. Filters suitable for the used fluorophores and a filter to detect autofluorescence were used. The Z-stacked images were transformed using the maximum intensity projection function in ImageJ v. 1.48 [82], to merge the signals into a one plane image. CellProfiler v. 2.2.0 [83,84] was then used to analyse the signals. The autofluorescence data were used to subtract background from the images, after which the images were cleared using a white tophat filter to remove anything over 10 pixels in diameter, leaving only the amplified signal. DAPI staining was used to define cells to enable automated counting of PLA signals within specific cells, and all signals with pixel intensity above 0.08 were automatically counted. The combined signal from all brain areas was divided with number of cells, to get an average of interactions within the brain. A graph was plotted using GraphPad Prism 5 software.

### 2.6. Analysis of gene expression after complete amino acid starvation in N25/2 mouse hypothalamic cells

It was previously shown that gene expression of *Mfsd11* is altered upon complete amino acid starvation for 1, 2, 3, 5 or 16 h in immortalized N25/2 mouse hypothalamic cells [60]. Here, we reused the data from their microarray analysis (accession number GSE61402) to study if the atypical SLCs were affected by the removal of all amino acids. Data were downloaded and the probes most similar to the human proteins were included in the analysis. Note that two genes (*Unc93a* and *Cln3*) had two probes each that correspond to the human protein on the GeneChip, which is why both are presented in the heat map. The duplicated probes are splice variants that are present under different accession numbers in the database used to define the genes on the chip. Genesis version 1.7.6 was used to generate the heat map. For 1, 2, 3 and 16 h, the difference between the log_2_ values of expression between starved and control cells were used in the analysis. For 5 h of starvation, the log_2_ fold change value of expression was used. Green colour represents downregulation and red colour represents upregulation, where more alteration correlates with more colour intensity.

## 3. Results

### 3.1. Interrelations between human SLCs of MFS type

**Figure 1.**
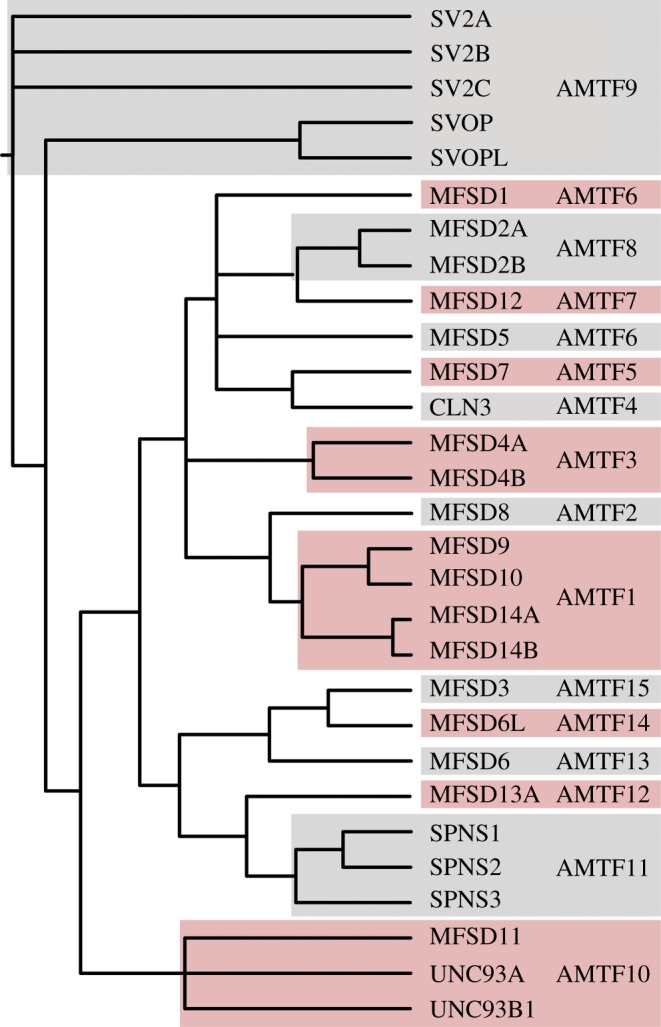
Interrelations between human atypical SLCs. The Bayesian approach was implemented when inferring the phylogenetic interrelations between the longest splice variants for 29 human atypical SLCs. When combining the phylogenetic clustering with sequence identities, the proteins could be divided into 15 families denoted Atypical MFS Transporter Family (AMTF) 1-15. The tree displays the schematic branching order of the human atypical SLCs of MFS type, together with CLN3.

The phylogenetic interrelations between atypical SLCs were inferred in the phylogenetic tree presented in figure 1, where the schematic branching order is displayed in the figure. Some sequences were seemingly diverged from the other proteins, like MFSD3, MFSD6, MFSD6 L, MFSD7, MFSD8, MFSD12, MFSD13A and CLN3 (figure 1), while others formed potential families connected by a common node. Grouping of proteins is important as it strengthens the possibility to elucidate evolutionary conservation, mechanism and substrate specificity, because similar sequences usually share these characteristics [85]. To divide the atypical SLCs into families, members had to share phylogenetic closeness and be 20% identical to other proteins in the family.

Among the atypical SLCs we identified 15 possible families that were denoted Atypical MFS Transporter Family 1-15 (AMTF1-15); where seven families contained more than one atypical SLC protein. AMTF1 included MFSD9, MFSD10, MFSD14A and MFSD14B; AMTF 3 contained MFSD4A and MFSD4B; and AMTF6 had MFSD1 and MFSD5 as members. MFSD2A and MFSD2B belonged to AMTF8; while SV2A, SV2B, SV2C, SVOP and SVOPL were in AMTF9. AMTF10 included MFSD11, UNC93A and UNC93B1; and AMTF11 consisted of SPNS1, SPNS2 and SPNS3 (figure 1). To examine the plausible family members further, similarities between protein sequences were analysed. All sequence identities were listed in the matrixes in supplementary table 1, where 24 of the 29 atypical SLCs had more than 20% identical amino acids to at least one other atypical SLC sequence. MFSD3, MFSD6, MFSD6 L, MFSD8 and MFSD13A had less than 20% identity with any other atypical SLC protein. In predicted AMTF1 (for members, see figure 1), all four proteins shared more than 20% identity with at least one other member, as were the case for AMTF9, AMTF10 and AMTF11. In AMTF3, MFSD4A and MFSD4B shared 20% identity, and AMTF8 was constituted by MFSD2A and MFSD2B sharing 37% identity. MFSD1 and MFSD5 did not cluster in closest proximity, yet shared 20% identity, and were considered constituents of the same family. The remaining eight atypical SLCs did not meet the clustering and/or identity criteria and were placed in individual families. Taken together, the atypical SLCs can be grouped into 15 possible AMTF (summarized in figure 1). The AMTF nomenclature was used instead of the SLC nomenclature to highlight that the functions of the atypical SLCs remains to be elucidated.

**Figure 2.**
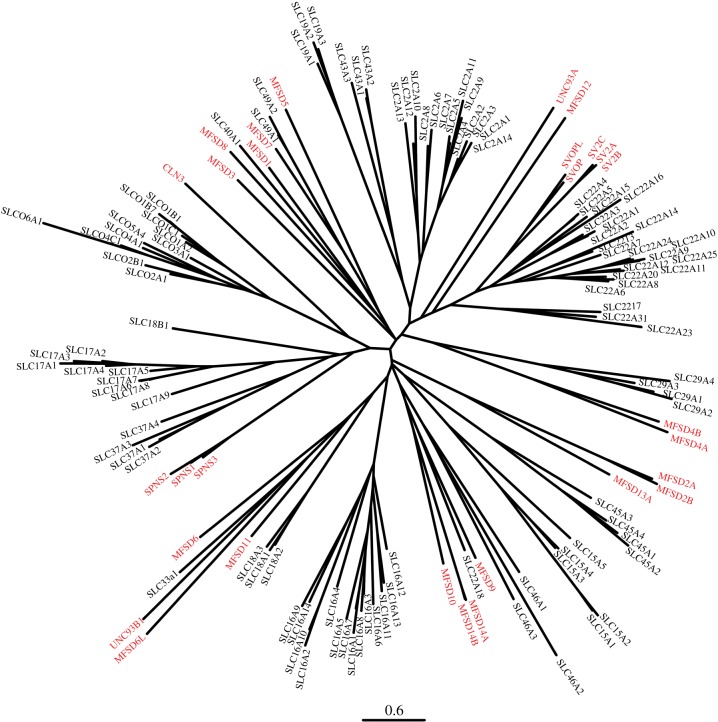
Atypical SLCs cluster among known SLCs of the MFS clan. RAxML was used to calculate a phylogenetic tree, showing how the atypical SLCs were related to known SLCs of MFS type. Trees were calculated on a model with 500 bootstrap replicas, and combined into a final tree using the built-in consensus tree calculation in RAxML. The highlighted proteins correspond to the atypical SLCs.

The distribution of the atypical SLCs among the SLCs of MFS type was investigated through a phylogenetic analysis. It showed that the proteins of interest placed within the SLC tree, and not as outgroups (figure 2). This strengthens the hypothesis that they are novel transporters of SLC type. When comparing the sequence identities (MFS matrix 2 in supplementary table 1), the following atypical proteins had less than 20% identity with any other SLC: MFSD2A, MFSD4B, MFSD6, SV2A, SV2B, SV2C and UNC93B1. On the other hand, some atypical SLCs had at least 20% identity to members of several families, like MFSD1, which was more than 20% identical with SLC2A8, SLC16A10, SLC19A2; and MFSD9 and MFSD10, having 20% or higher identity with members from seven different SLC families each. Finally, no atypical SLC shared more than 20% with all members in a single SLC family. Therefore, it is not possible to place the atypical SLCs into existing SLC families based only on sequence identity. However, when combining the sequence identity and phylogenetic clustering (figure 2), possible family clustering is observed; MFSD7 (in AMTF5) is already classified as a member of SLC49 [25], while MFSD9, MFSD10, MFSD14A and MFSD14B (AMTF1) could belong to SLC46, and SV2A, SV2B, SV2C, SVOP and SVOPL (AMTF9) could be members of SLC22. The remaining atypical SLCs would belong to novel SLC families. If we combine the 52 SLC and 15 AMTF families (where AMTF1 is merged with SLC46; AMTF5 with SLC49; and AMTF9 with SLC22), a total of 64 different families including SLC proteins exists.

### 3.2. Identification of related proteins in several species

**Figure 3.**
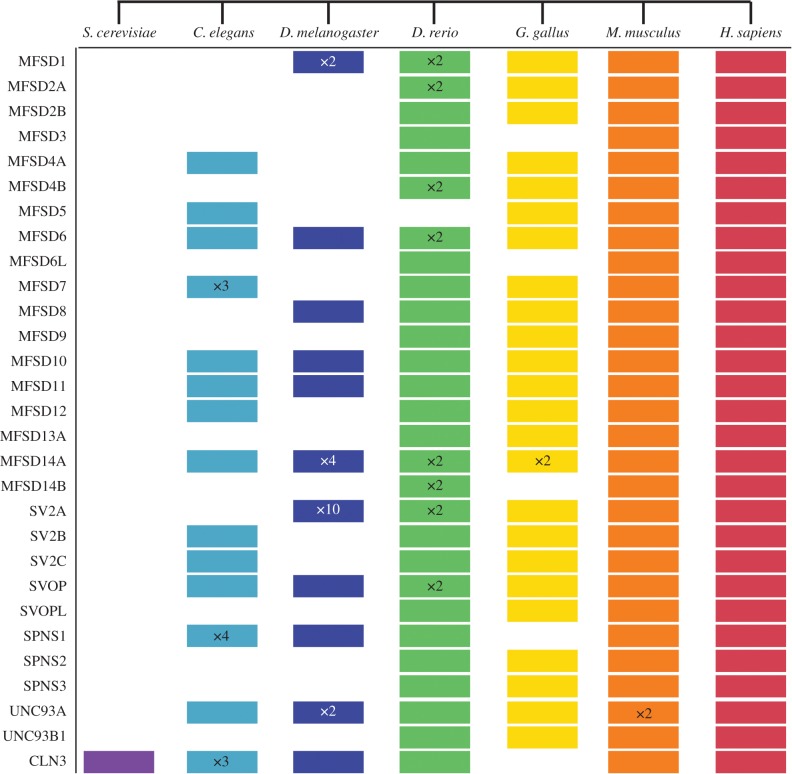
Evolutionarily conserved proteins. Hidden Markov models were used to identify related proteins to the atypical SLCs in the listed species. In this schematic description the coloured cartons indicate the presence of a related atypical SLC, while no box indicates a missing protein. The × *n* designation corresponds to the amount of proteins/variants identified, where *n* is a specific number. No specific marking was made where only one variant was found.

With hidden Markov models, several protein datasets were searched to identify related proteins in various species. The atypical SLCs were identified in human and mouse (figure 3), where UNC93A had duplicated in mouse resulting in two variants on the same chromosome. All but MFSD3, MFSD6 L, SPNS1 and CLN3 were found in chicken (figure 3). Furthermore, MFSD14B was identified in both the MFSD14A and MFSD14B HMM search in the chicken proteome, but it phylogenetically clustered closer to human MFSD14A. Therefore, MFSD14B was not separately included in figure 3 or electronic supplementary material, table S2, but as one of the two proteins found for MFSD14A. All except MFSD5 were detected in zebrafish (figure 3). Eight proteins had two copies each in the zebrafish proteome. 11 atypical SLCs had related proteins in fruit flies (figure 3), where MFSD1 had two copies, MFSD14A had four copies (equally related to MFSD14B), SV2A had 10 (equally related to SV2B and SV2C) and Unc93A had two copies (equally related to UNC93B1). In the figure, we enlisted the proteins where they were most similar, and if they were equally related to several proteins we listed them in the first possible position. Identified proteins were sometimes found in several HMM, but they were included only once in figure 3 and electronic supplementary material, table S2. About half of the atypical SLCs were found in *C. elegans*, while only CLN3 was identified in yeast. Furthermore, in some proteomes, several related proteins were found but they did not cluster phylogenetically with the human proteins, but still in relative proximity. We call these ‘Like’ (L) proteins, and they are included in electronic supplementary material, table S2, but not in figure 3. There are, for example, 11 proteins related to MFSD8, but none in the human cluster, and they were annotated as MFSD8L1–MFSD8L11.

### 3.3. Atypical SLCs are predicted to have 12 TMS

**Figure 4.**
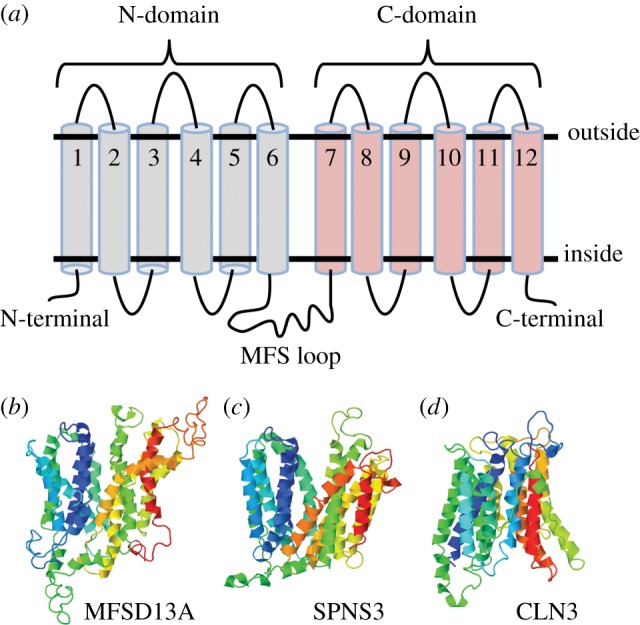
Structural prediction of the atypical SLCs. The online tool CCtop [73] was used to predict the topology of the atypical SLCs, where all but three proteins were predicted to possess the N and C domain, connected by a long cytoplasmic loop (MFS loop), resulting in a 12 transmembrane segment (TMS) polypeptide, as schematically depicted in (*a*) MFSD13A, SPNS3 and CLN3 diverged from the common structure, for which homology models were built to verity the predictions. The three proteins were aligned against structurally known MFS proteins, using the automated Swiss model homology program [77]. MFSD13A (*b*) and SPNS3 (*c*) were both constituted by 12 TMS, while CLN3 (*d*) had 11TMS. All three contained the long intracellular loop between TMS6 and 7.

We used CCtop to predict the structural appearance of the human atypical SLCs. All but MFSD13A (9 TMS), SPNS3 (11 TMS) and CLN3 (11 TMS) were predicted to contain 12 TMS, the common number for MFS proteins [17]. Six TMS has been suggested for CLN3 [31,32,86,87], but different TMS has been found by the different groups. The general 12 TMS structure is schematically depicted in figure 4*a*. MFSD6, SV2A, SV2B, SV2C and UNC93B1 were seemingly longer peptides than the regular MFS peptide (table 1), and they all were predicted to contain exceptionally long N-terminals. Furthermore, MFSD6 had a relatively long extracellular loop between TMS 3 and 4, while the SV2 proteins had a longer loop between TMS 7 and 8. To verify the structure of the irregular predictions of MFSD13A, SPNS3 and CLN3, homology models were built. Structurally known MFS proteins were used as templates. In the homology models both MFSD13A (figure 4*b*) and SPNS3 (figure 4*c*) were predicted to contain the expected 12 TMS, whereas CLN3 (figure 4*d*) still was composed of 11 TMS. When manually comparing the amino acids in each TMS that were identified in CCtop versus the homology models, it was revealed that MFSD13A consisted of several amphipathic TMS (figure 4*b*), which could explain why they were not identified by CCtop. For SPNS3, all TMS overlapped, except TMS11, which was lacking in the secondary structure prediction. As TMS 11 was amphipathic, it could have been considered as a too short hydrophobic segment to be identified as a TMS by the CCtop server. Finally, for CLN3, both models predicted the same TMS. In conclusion, we predict all studied atypical SLCs to have 12 TMS, except CLN3, which was predicted to have 11 TMS.

### 3.4. Several atypical SLC genes are expressed in the same cells

**Figure 5.**
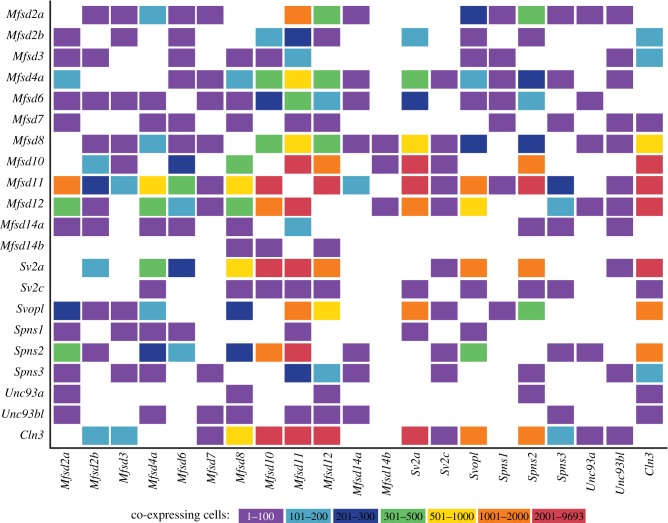
Single cells co-express atypical SLC transcripts. Single-cell RNA sequencing data from a mouse embryo brain was retrieved from 10X Genomics, and analysed to study co-expression of atypical SLC genes in single cells. Twenty-one atypical SLCs were co-expressed in various combinations in 9693 cells. In the figure, boxes represent co-expression, where colours represent number of co-expressing cells. Purple correspond to 0–100 co-expressing cells, light blue 100–200 cells, dark blue 201–300 cells, green 301–500 cells, yellow 501–1000 cells, orange 1001–2000 cells and red represents more than 2001 co-expressing cells.

To study co-expression of atypical SLC genes in embryonic mouse brain cells, data from single-cell RNA sequencing was analysed. Co-expression of at least two atypical SLC transcripts was identified in 9693 of the total 10 289 cells analysed. Twenty-one of the atypical SLCs were found as significantly co-expressed with other atypical SLCs (figure 5). *Mfsd1*, *Mfsd4b*, *Mfsd5*, *Mfsd6l*, *Mfsd9*, *Mfsd13a*, *Sv2b* and *Svop* were not detected in the analysis, probably due to the relatively shallow sequence depth or utilized cut-off values. There are three different *Mfsd7* (*Mfsd7a-c*) genes in mice corresponding to human *Mfsd7*, but only *Mfsd7c* was found in the dataset. Some genes were co-expressed with several other genes, like *Mfsd11*, which was co-expressed with all studied atypical transcripts except *Mfsd14b* and *Cln3*. Others showed more stringent co-expression, like *Mfsd14b*, which only co-localized with *Mfsd8*, *Mfsd10* and *Mfsd12*. The sequentially similar *Mfsd2a* and *Mfsd2b* displayed a complementary co-expression, and together they were co-expressed with all found atypical SLCs except *Sv2a* and *Sv2c*. The three *Spns* genes supplemented each other, and together they were expressed in the same cells as all other genes except *Mfsd14b* (figure 5). Regarding AMTFs, *Mfsd10* showed extensive co-expression with 12 other genes, while its family member were more restricted; *Mfsd14a* was co-expressed with eight other genes and *Mfsd14b* with only 3, while *Mfsd9* was not detected at all. Some of the co-expressions were found only in few cells, like *Unc93a* having only 1–2 cells containing each interaction (figure 5). Among the more frequently found co-expressions were *Cln3* together with *Mfsd10*, *Mfsd11*, *Mfsd12* or *Sv2a*, with co-expression in more than 3000 cells (figure 5).

**Figure 6.**
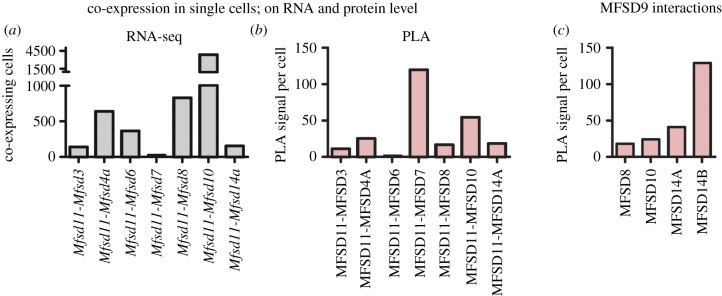
Verification of co-expression at protein level. *In situ* PLA was run on mouse brain sections to study interaction between certain atypical SLC proteins, to verify the single-cell RNA sequencing. (*a*) *Mfsd11* was co-expressed with several atypical SLCs using the RNA sequencing dataset. (*b*) The co-expression of the corresponding proteins was also detected at protein levels using *in situ* PLA. Some atypical SLCs were not detected in the single-cell RNA sequencing data, likely due to low transcript detection. However, interactions for those proteins were still found using *in situ* PLA. (*c*) Protein–protein interactions detected by PLA between MFSD7, which was not found on transcript level, and its closely related proteins MFSD8, MFSD10, MFSD14A and MFSD14B are shown here.

To supplement the co-localization and to detect probable interactions at protein level, *in situ* proximity ligation assay was run. As *Mfsd11* was most commonly found as co-expressed on transcript level (figure 6*a*), a subset of its combinations were selected and tested. In all selected combinations, interaction signals were identified, but at different degrees, confirming that co-expressed RNA transcripts were found at protein level (figure 6*b*). Even genes such as *Mfsd9*, which was not found to be co-expressed in the RNA sequencing, was found in proximity to other atypical SLCs at protein level (figure 6*c*).

### 3.5. Transcriptional changes upon amino acid starvation

**Figure 7.**
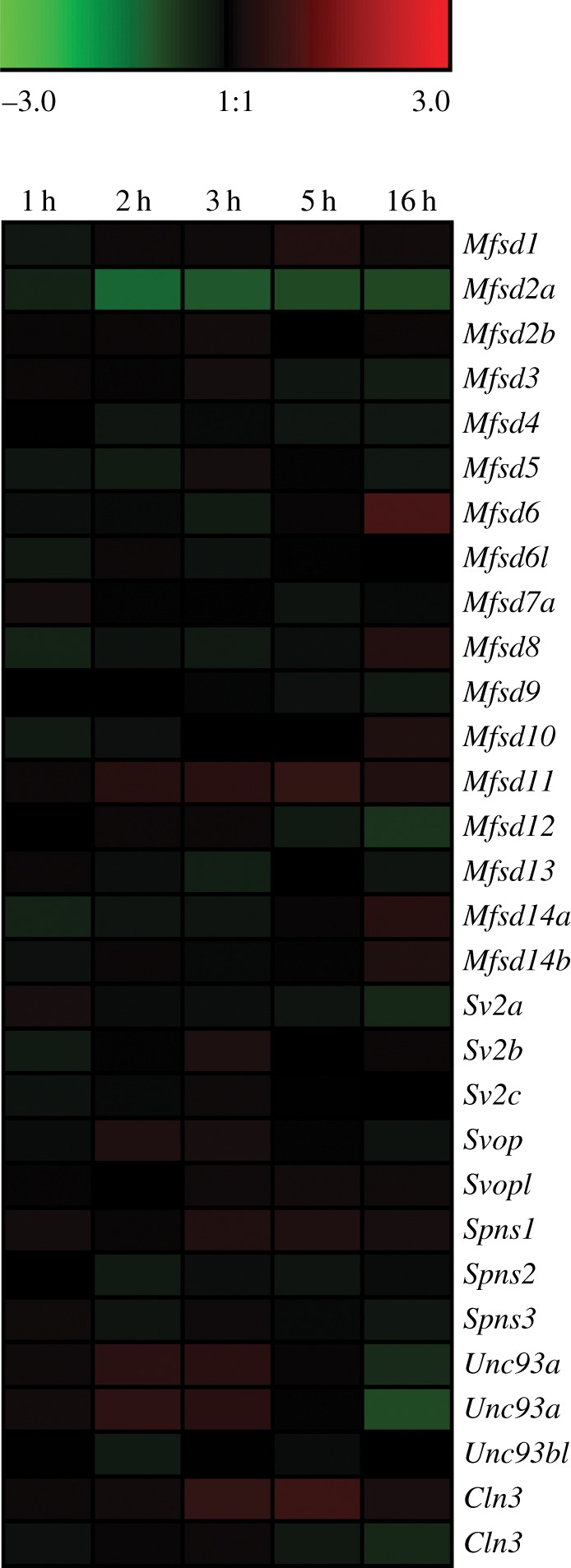
Transcription levels of atypical SLCs are changed upon complete amino acid starvation. Mouse hypothalamic N25/2 cells were deprived of all amino acids for 1, 2, 3, 5 and 16 h, followed by microarray analysis to study transcriptional changes [60]. Data accession number was GSE61402. Genesis version 1.7.6 was used to generate the heat map, which depicts log_2_ difference between starved and control cells at each time point. Green colour depicts downregulation while red colour corresponded to upregulated expression, where larger changes correlate with stronger colour intensity. Note that for *Cln3* and *Unc93a*, two probes were identified corresponding to the human proteins, and both were included in the analysis.

Mouse hypothalamic N25/2 cell lines were deprived of amino acids for 1–16 h, followed by gene expression analysis. The alterations in gene expression for the atypical SLCs were depicted in a heat map (figure 7), with corresponding log_2_ differences listed in table 4. All genes were affected at all times, except *Mfsd6l* after 16 h, *Mfsd9* after 1 h and *Mfsd13a* after 5 h. *Mfsd2a* and *Spns2* were reduced throughout the experiment, whereas *Mfsd11* and one of the *Cln3* reported increased expression (figure 7). *Mfsd8* was reduced up to 5 h, after which the expression was enhanced. The opposite pattern was seen for both *Unc93a* probes on the array, with upregulation during the first 5 h, followed by reduction after 16 h (figure 7). The duplicated probes for *Unc93a* and *Cln3* on the array are probable splice variants listed under different accession numbers, and the pairs of probes follow the same trend in expression change. At 5 h, adjusted *p*-values were calculated, showing significant reduction of *Mfsd2a* (adj. *p* = 0.00041), while the *Mfsd1* (adj. *p* = 0.0029)*, Mfsd11* (adj. *p* = 0.00003) and one *Cln3* (adj. *p* = 0.00007) genes were upregulated (adjusted *p*-values listed in table 4).

**Table 4.** Results from amino acid starvation on N25/2 mouse hypothalamic cells [60]. Asterisk indicates significantly changed expressions.

gene	probe ID	log_2_ 1 h	log_2_ 2 h	log_2_ 3 h	log_2_ 5 h	adj. *p*-value (5 h)	log_2_ 16 h
*Mfsd1*	10492499	−0.17	0.11	0.12	0.34	0.00290*	0.15
*Mfsd2a*	10516064	−0.36	−1.21	−1.01	−0.86	0.00041*	−0.85
*Mfsd2b*	10399314	0.08	0.09	0.16	−0.01	0.97988	0.09
*Mfsd3*	10424991	0.09	0.05	0.18	−0.15	0.25122	−0.26
*Mfsd4a*	10357660	0.02	−0.14	−0.05	−0.13	0.26274	−0.17
*Mfsd5*	10427162	−0.14	−0.23	0.18	0.04	0.76622	−0.17
*Mfsd6*	10354506	−0.07	−0.05	−0.23	0.06	0.69534	0.91
*Mfsd6l*	10377308	−0.19	0.10	−0.10	0.02	0.92248	0.00
*Mfsd7a*	10532169	0.19	−0.03	0.03	−0.11	0.58350	−0.05
*Mfsd8*	10497944	−0.36	−0.10	−0.20	−0.08	0.57402	0.36
*Mfsd9*	10354220	0.00	−0.01	−0.04	−0.09	0.48358	−0.21
*Mfsd10*	10529410	−0.20	−0.08	0.01	−0.01	0.94898	0.33
*Mfsd11*	10382852	0.10	0.41	0.47	0.65	0.00003*	0.34
*Mfsd12*	10365104	−0.01	0.10	0.10	−0.21	0.16511	−0.62
*Mfsd13a*	10463632	0.09	−0.08	−0.30	0.00	0.99228	−0.12
*Mfsd14a*	10501676	−0.36	−0.13	−0.12	0.07	0.68940	0.44
*Mfsd14b*	10410173	−0.09	0.09	−0.05	0.04	0.86899	0.33
*Sv2a*	10494372	0.21	−0.07	−0.08	−0.12	0.38332	−0.42
*Sv2b*	10564646	−0.21	−0.03	0.29	−0.01	0.95483	0.09
*Sv2c*	10411274	−0.10	−0.05	0.12	0.02	0.93726	0.01
*Svop*	10532784	−0.06	0.30	0.21	0.04	0.85594	−0.09
*Svopl*	10544017	0.05	0.01	0.12	0.15	0.19576	0.13
*Spns1*	10567838	0.17	0.08	0.34	0.30	0.04250	0.20
*Spns2*	10388194	−0.02	−0.22	−0.08	−0.12	0.40316	−0.07
*Spns3*	10388211	0.13	−0.12	0.12	−0.05	0.81915	−0.16
*Unc93a*	10447634	0.12	0.53	0.48	0.06	0.89752	−0.50
*Unc93a*	10447904	0.16	0.57	0.48	0.04	0.92946	−0.88
*Unc93b1*	10460237	0.02	−0.21	0.01	−0.07	0.61249	−0.02
*Cln3*	10557434	0.11	0.15	0.65	0.78	0.00007*	0.24
*Cln3*	10567964	−0.09	0.08	0.11	−0.18	0.12644	−0.41

## 4. Discussion

Here we investigated the characteristics of 29 novel predicted transporters, denoted atypical SLCs, to get a comprehensive understanding of their phylogenetic interrelations, family clustering, protein structures, co-expression and how they responded to altered amino acid levels. With phylogenetic trees, we elucidated the interrelations between the atypical SLCs alone, and how they group among the known SLC of MFS type. Upon closer inspection, the two phylogenetic trees provided mostly similar results, but not identical. UNC93A, for example, clustered with MFSD11 and UNC93B1 in figure 1, and closest to MFSD12 in figure 2. The reasons for this discrepancy could be several. First, we used different programs for tree calculations. MrBayes is a good tool concerning small-to-medium alignments, but for larger and more complex datasets, other methods, like the likelihood method implemented in RAxML [69], have to be used. Here, the main reasons for differences are within the tree searching algorithms. With the more advanced and computational intensive models implemented in MrBayes, it will be possible to investigate a smaller proportion of the total number of possible trees compared to RAxML. In addition, the more stringent models implemented in MrBayes will not converge in reasonable time for more complex datasets. Second, as more sequences were included when compiling figure 2, there were larger variations, resulting in a less accurate starting alignment. This is why the tree in figure 1 was considered most accurate and primarily used for family clustering, while the second figure showed that the atypical SLCs cluster with SLCs.

The atypical SLCs are probably SLC proteins, but most are still orphan regarding function. Therefore, they were divided into AMTF families instead of using the existing SLC nomenclature. This highlights that the proteins are possible transporters, but that their function remains to be elucidated. Whenever their functions are determined they can be renamed according to the SLC root system, which could result in 64 SLC families instead of the present 52 SLC families.

In general, proteins within a SLC family usually share mechanism and substrate profiles [85], although exceptions to this rule can be observed. Most proteins in the AMTFs are not well studied, but there seem to be both similarities and differences within the families. AMTF1 (MFSD9, MFSD10, MFSD14A and MFSD14B) and AMTF8 (MFSD2A and MFSD2B) are examples for similarities and dissimilarities. In AMTF1, MFSD10 is identified both at the plasma [47] and intracellular membranes [36], while MFSD14A and MFSD14B have only known intracellular expressions [33]. MFSD8, which shares a branching node with the AMTF1 proteins, is also intracellular [27]. Therefore, it is likely that MFSD9 also has an intracellular location. This hypothesis was strengthened as we detected interaction between MFSD9 and MFSD8, MFSD10, MFSD14A and MFSD14B using *in situ* PLA. This means that MFSD9 is located within 40 nm proximity of the other three intracellular proteins. Regarding their substrates, they are believed to differ as MFSD10 transport organic ions [36], while MFSD14A is suggested to be sugar transporter as it shares several structural characteristics with known sugar transporters [48]. MFSD14B is a predicted sugar transporter due to its high sequence identity (67.7%) to MFSD14A. However, similar response patterns to amino acid deprivation were found, where small changes were detected until 5 h for all four members, followed by upregulation of all but *Mfsd9* after 16 h.

If we instead consider AMTF8, both MFSD2A and MFSD2B are located to the endoplasmic reticulum [37], while MFSD2A is also detected in the plasmalemma [42]. As they are nearly 40% identical, it is likely that they share a substrate and mechanism, and as MFSD2B transports lipids in a sodium-dependent manner [43], it is possible that MFSD2B does so as well. The genes were expressed together in some cells, and their combined transcripts were found with all atypical SLCs, except the *Sv2s*, suggesting they could have similar effects. *Mfsd2a* was co-expressed with 14 atypical SLCs, while *Mfsd2b* co-expressed with 12 genes, of which they shared co-expression with 7 genes. This suggests that MFSD2B could function as the back-up system for MFSD2A in specific cells or that it may have a more direct and specific function. They responded differently to amino acid starvation, where *Mfsd2a* was significantly reduced, while *Mfsd2b* remained unaffected. It is possible that *Mfsd2b* functions as a housekeeping gene, and hence lacks alteration upon diet change. On the other hand, *Mfsd2a* could have a direct function in energy balance, and is therefore found to be affected by starvation. Taken together, there are both similarities and differences between AMTF members, and it is not yet possible to elucidate their expression or functions, but the family clusters are good suggestions on which further investigations can be based.

To understand how single cells maintain their homeostasis, preserve ion balances, keep optimal sugar levels and so on, we must figure out which transporters are expressed together. By studying single-cellular transcriptomes, we identified genes that seem to be co-expressed with several other atypical SLCs, like *Mfsd8*, *Mfsd11* and *Mfsd12,* suggesting that are needed for basic maintenance, while other genes displayed a more restricted co-expressions, like *Mfsd14b* and *Unc93a*. In the RNA sequencing analysis, there were approximately 42 000 reads per cell, meaning that low-expressed genes are probably missing from the dataset. This is why undetected but anticipated co-expressed transcripts, like *Mfsd9*, could still be found as interacting partner to other proteins *in vitro*. There were detectable PLA signals even though the corresponding genes were not present in the sorted RNA dataset. This can be explained by the fact that low levels of mRNA can result in high protein translation in mammalian cells [88]. In many cases, mRNA and protein levels do not correlate completely because of different regulation controls. From the experiments we conclude that if genes were co-expressed according to the RNA sequencing, they were indeed found in the same cell. However, we cannot deduce anything about the unfound interactions; even if *Mfsd2b* has fewer gene co-expressions than *Mfsd2a*, transcripts could have been missed. For the *in situ* PLA, interactions were considered as accurate and as confirmations of co-existing proteins in the same cell, but comparisons between protein combinations were not performed. If we were able to understand the complete transporter co-expression map, it would facilitate the understanding of pharmacokinetics and human diseases.

Most MFSs are similar in structure [17], despite their relatively low sequence identities. Therefore, we found it convincing that the predictions of atypical SLCs containing 12 TMS were accurate. This was in accordance with previous publications describing the structure of some atypical SLCs based on other topology prediction tools [27,29,30] or homology models [19,26]. As the predictions for MFSD13A, SPNS3 and CLN3 did not support our hypothesis, we built homology models to verify their predicted structures. When building homology models, the sequences were aligned against a structurally known MFS protein, providing higher reliability to the model than the prediction pool based only on amino acid sequences. This is why we feel confident to suggest that MFSD13A and SPNS3 have 12 TMS each. Interestingly, we identified only 11 TMS for CLN3 using CCtop and homology modelling, while previous reports have postulated conflicting results [31], where a six TMS protein is seemingly accepted [31,32,86,87]. However, it is different six TMS that are predicted in previous publications [31]. We have identified all previously predicted TMS, and additionally two regions, TMS 8 and 11, which have not been suggested so far. To our knowledge, no homology models have previously been built for CLN3. Since it does not belong to any Pfam clan, but is a clustered as member of the MFS superfamily according to the Transporter classification database [24], and because it shared between 10 and 20% sequence identities with many MFS proteins, we decided to align it against an MFS template. As the predicted TMS corresponded with those found by CCtop we considered it as a reliable three-dimensional model. Therefore, we deviate from previous reports, and propose an 11 TMS structure for CLN3. Among SLCs belonging to other Pfam clans, 11TMS is a common structure (e.g. the SLC38 family belong to the APC Pfam clan, and they all are predicted to contain 11 TMS [89]). It is thus possible for an atypical SLC to have such structure.

Since the atypical SLCs phylogenetically group among SLCs of MFS type, share the MFS transporter topology and are affected by complete amino acid deprivation in cell cultures, it is likely that these proteins are novel transporters. As there has been a call for systematic research on transporters [6], we suggest that the atypical SLCs should be included in this. They could interact with drugs and be associated with diseases.

## Supplementary Material

Sequences and their shared identities

## Supplementary Material

Proteins related to the atypical SLCs

## Supplementary Material

Abbreviation list

## Data accessibility

All data are available through the main article and the online electronic supplementary material.
